# Supplementary material for: Correlation between preoperative uric acid levels and lymph node metastases in patients with papillary thyroid cancer: a retrospective study
Source: PeerJ. 2025 Jul 10;13:e19410. doi: 10.7717/peerj.19410 (PMC12256038; doi:10.7717/peerj.19410)
Supplement: Supplemental Information 2 [file peerj-13-19410-s002.docx]

**Codebook**

CLNM: 0= without central lymph node metastases ; 1= central lymph node metastases

Sex: 0=male ; 1=female

UA: μmol/L

TG: mmol/L

LDL-C: mmol/L

Cr: μmol/L

Glu: mmol/L

FT3: pmol/L

FT4: pmol/L

TSH: mIu/L

Braf: 0=without BRAF gene mutation (V600E) ; 1= BRAF gene mutation (V600E)

Age: 0= Less than 55 years old; 1= Greater than or equal to 55 years old

Tumorsize: 0= Less than or equal to 0.5cm; 1= Greater than 0.5cm

CLNMnumber: Number of lymph node metastases

Facility：0= Unifocal; 1= multifocal

Capsularinvasion: 0=without extrathyroidal infiltration; 1=with extrathyroidal infiltration

HT: 0=without history of Hashimoto’s thyroiditis; 1=with history of Hashimoto’s thyroiditis

Location: 0= Tumor location at left thyroid gland lobe; 1= Tumor location at right thyroid gland lobe; 2= Tumor location at the isthmus of the thyroid gland; 3= Tumor location at bilateral thyroid lobes

Margin：0= Smooth; 1= Ill-defined

Shape：0= Oval; 1= Taller-than-wide

Echo：0= Hypo echogenicity; 1= Iso/Hyperechogenicity

Miccal：0=without microcalcification; 1=with microcalcification
